# Supplementary material for: Strategic rule breaking: Time wasting to win soccer games
Source: PLoS One. 2019 Dec 18;14(12):e0224150. doi: 10.1371/journal.pone.0224150 (PMC6919576; doi:10.1371/journal.pone.0224150)
Supplement: Attachment — Submitted filename: PLOSreplyFinal.docx [file pone.0224150.s001.docx]

*Reviewer #1: Soccer is going through its own “Moneyball” moment, as teams make use of extraordinarily detailed data on player movements and game play to optimize their strategies, see e.g., “The Numbers Game” by Chris Anderson and David Sally. To the best of my knowledge, this is the first paper in the management-science literature to use that same data.

The authors consider stoppages (due to throw-in, free kick, goalie restart, etc) and show convincingly that teams that are leading the game tend to take more time to restart while teams that are losing take less time to restart, compared to when the score is tied. Moreover, the magnitude of these effects is substantial. For instance, when throwing in the ball, teams that are leading take about 15% more time than teams that are tied, who in turn take about 15% more time than those that are losing. This is interesting.

Unfortunately, I have three major concerns with the paper that make it unsuitable for publication in its current form.

FIRST, the authors’ key conclusions do not follow from their findings. “We predict that a change to rules with stopped game clock when the play is stopped would make game play more time efficient” (abstract). This would be true if “strategic time-wasting” was the only effect at play but, as noted above, teams that are losing ALSO respond to their incentives, speeding up play in their urgency to score before time runs out. So, any time savings that one would get by speeding up the leading team will be counteracted by slowing down the trailing team. Indeed, to the extent that the trailing team benefits more from resting its players—to be capable of a few last bursts of athleticism to score—we might expect the losing team to delay even MORE than what the analysis here suggests. Overall, then, it is unclear whether stopping the clock would reduce or increase the amount of time wasted overall. Certainly, it is NOT true that “The empirical results and simulations offer clear advice to the soccer governing bodies” (conclusion).

To resolve this first concern, the authors should provide a balanced analysis that emphasizes how the current rules incentivize both time-wasting and time-saving. They could then discuss how switching to a stopped clock would have mixed effects (speeding return to play in some cases, slowing it in others) that need to be carefully weighed.*

We agree that our presentation of the findings was not sufficiently balanced. We knew that the time-wasting exceeded the speeding-up in total but did not quantify this. We have now done so, and we present the findings in a new paragraph (p. 10). As we report, only for corner kicks is there a net gain from the sum of time wasting and speeding-up; for all other outcomes stopping the clock saves time, though by a trivial amount for throw-ins. Indeed, the IFAB/FIFA reaction to our findings was that they knew about the overall slowing-down effect of the current rule system, but they were shocked to see that the level of cheating was so systematic and its effect so large.

Also, please note that speeding-up of the start of play because the clock runs is not seen as a benefit by the governing bodies because it means that at least some re-starts are done too early, with players of the starting team not in good position for the subsequent play. Although quick starts entertain the audience, they are contrary to the fair-play norm.

By the way, the conjecture of “strategic resting” for a later burst (resulting in higher total probability of scoring) is not seen in the data. On the contrary, the scoring rate is higher towards the end of the game, which would be consistent with there being a possible secondary effect of making the other team more tired in order to increase the possibility of another goal. (This would have effects both ways, but if the trailing team is the one that scores, it is good. But losing with 2 goals is not much worse than losing with 1 goal.)

*SECOND, the authors’ discussion of causation of patterns found in the data is questionable and likely to confuse readers who are unfamiliar with causal reasoning. For example: “Teams that are ahead get fewer opportunities to throw the ball in, consistent with the opposing team seeking to deny them this time-wasting opportunity.” The authors here are suggesting a fascinating possibility, that trailing teams might be intentionally keeping the ball in bounds, to stop leading teams from wasting time. But the paper provides no causal analysis to support this possibility. This could leave readers with the wrong impression that the conjectured causal explanation has been shown to be true. (Experts understand that the phrase “consistent with” is code for “we have no idea if this is true”, but this paper could reach a broader audience of soccer enthusiasts who are not as familiar with the terminology.)

This second concern can be resolved by dropping all causal speculation or, if the authors want to discuss potential explanations, to be sure to include at least two alternative explanations for each pattern discussed.*

We agree with this observation and have made our writing more cautious. Please examine the deletions and rewriting in our discussion of Fig. 1, as well as our writing elsewhere.

We should note, however, that this research started based on conjectures made by football professionals. (One of these is the brother of one of the authors, who played professional soccer for 17 years, including English Premier League, Italian Serie A and 46 games for the national team, and since ending the playing career has worked for 12 years as a player scout and soccer analyst.) The language we toned down in this revision was already toned down based on the observations that inspired the research. For example, defending players of a team that is behind definitely seek to keep the ball in play rather than allowing throw-ins, unless they can ensure that the throw-in is also a turnover of ball control. Figure 1 shows that they are successful.

*THIRD, the “rational model” is not really a “rational model”. The **value** of delay is introduced in line 185 and corresponds to the extra likelihood of winning or tying when there is less time remaining (which, of course, depends on the current state of the game, team characteristics, etc). However, there is no **cost** of delay! (In the case of slowing down by a leading team, the benefit is that having less time remaining improves odds of winning, while the cost is that being penalized decreases odds of winning. In the case of speeding up by a trailing team, the benefit is that having more time improves odds of tying, while the cost is that rushing may lead to a poorer re-start to play.) Moreover, the extent of these costs are undoubtedly non-linear in the amount of delay, e.g., referees may be more likely to give a yellow card as more time passes without a restart of play. (The authors appear to obliquely reference cost in lines 195-196 “The split variable will capture whether teams have different **ease** of speeding up and slowing down the game.” However, I find this very confusing since the “split variable” is referring to different “parts” of the value of a minute, meant to capture “the different effects for delaying or speeding up the game.” What does that mean??)

The term “rational” refers to a choice being made optimally in pursuit of some objective. To study a “rational” actor, one must therefore fully specify the objective that is being maximized. For instance, to capture the objective of the leading team and hence have a truly rational model of delay here, one must not only estimate the “value of a minute” (as done, capturing the benefit of delay) but also the “cost of a yellow card” and “likelihood of a yellow card” (capturing the cost of delay).

This critique may seem like semantics, but there is important substance to it as well. With a rational model, one can explore and make predictions about what would have happened if the game had been changed. For instance, what if soccer’s governing bodies decided to encourage referees to increase the frequency with which they give yellow cards for delay. Armed with a rational model, one could conduct the relevant counterfactual analysis and produce quantitative estimates about the likely impact of such an intervention. By contrast, based on this paper’s analysis, you could say that there will be less delay—but have no way of quantifying how much less.

This concern could be addressed by motivating the writeup and framing the analysis more accurately, namely, as a calibration exercise, e.g. asking: “How much does delay increase or decrease winning chances [i.e. what is impact of delay]?”, “How much do teams respond per extra-% of winning by delaying the return to play [i.e., how sensitive are teams to the impact of delay]?”, and “Is each team’s sensitivity consistent across contexts, i.e., it is similar for throw-ins as for free kicks?”*

We are aware of the omission of the yellow-card risk from our model, and we chose not to incorporate it because of the difficulty of measuring it. Soccer teams are sufficiently strategic that a player who has a yellow card already would not expose himself to time-wasting yellow card risk, so the risk of becoming a 10-main team as a result of time-wasting is effectively zero. However, league play also involves counting yellow cards across games and making a player with too many cards ineligible for one game later in the season, so the effect of a time-wasting yellow card isn’t zero either. It is impractical to try to quantity it, so we left it out. If this makes the model insufficiently rational, we can change the naming to a value-based model. This naming is accurate because it precisely measures the value of a minute.

You have persuaded us that this renaming of the analytical model is necessary, and we think it is also a sufficient improvement of the paper. We thought about rephrasing the findings, such as through examining the effect on win probabilities or same-team consistency across actions, as you suggested. We think the current phrasing comes is close to our motivation for doing this analysis, as well as to what we have seen in soccer journalism and heard from IFAB/FIFA, so we believe it may have general appeal.

*Switching now from concerns to suggestions, here is an idea for a different sort of policy intervention: Introduce a new penalty “PURPLE CARD” that is issued specifically for delay in the latter part of the game (when strategic delay is most likely) and, when issued, gives the coach of the other team the option to extend game-play for one extra minute per purple card issued. A player on the leading team who drags his feet a few extra seconds making a throw-in now risks giving the other team one whole minute of additional time. Such a policy would keep the excitement of the trailing team’s urgency to get the ball back into play as soon as possible, while also reducing the leading team’s incentive to delay the game. (Another advantage of such a rule change is that it could be easily scaled, by adjusting the stringency with which referees enforce the rule. By contrast, eliminating clock stoppages is a dramatic and irreversible change—or, if it is reversed, highly embarrassing for the governing body!!)*

This is an interesting suggestion, and one that could be entertaining for the audience in addition to giving some more discipline to the time use. The main focus of IFAB/FIFA is to make the game fairer, however, and as a part of that remove opportunities to cheat and to facilitate objective decision making (Video Assisted Refereeing is a major initiative towards this). The current subjective mechanism to deal with time wasting is extra time awarded by the referee, which we can show to be pretty much random (i.e., the correlation with actual loss of time during the game is 0.048). IFAB/FIFA knows this and views referee judgment as having limited value in controlling time wasting. Given the current focus of the IFAB/FIFA PURPLE CARD would be unlikely to be considered because it relies too much on the subjective judgment of the referee.

As you note, the change in time management currently considered by IFAB/FIFA and informed by our analysis is indeed dramatic, and it is motivated by a growing concern that time wasting is a significant problem calling for a solution. Analytical results like these help the decision, but the governing bodies also have a well-established system for trying out rule changes at lower level leagues and tournaments before making them universal, as they did for VAR. They certainly have the resources necessary to do so. For example, the Bundesliga has 18 teams, but Germany has a total of 26,000 football clubs, many of which have multiple teams (men, women, age-delimited).

*Smaller comments

1. Line 69-70: Please check the sentence after “Corners show the same pattern”. Does “less” need to be switched to “more”? The pattern described appears to be the opposite of the pattern for free kicks.*

We have simplified the language in this paragraph. Corners and free kicks both show South-East movement going rightward in each column, so qualitatively they are similar. The main difference is not seen in the graphs but in the analysis, as the time-saving (South) movement is stronger there for teams that are behind.

*Reviewer #2: The paper is done responsibly, though I have a couple comments.

1) I'm not sure rational vs. non-rational is the right terminology. I know the spirit of rational expectations is that rational means forward looking, but this applies in specific contexts like asset pricing. Rational behavior could mean using past or current information in this context.*

Your comment made us reconsider the naming of this model and change it. We agree that our calculations are not exactly rational expectations because the cost (mainly the risk of getting a yellow card) is not included, and we have renamed it a value-based model. This language captures how the calculations were done because the model exactly measures the value of a minute. Similarly, the semi-rational model is now a semi value-based model. We think this language is descriptive of the content of each model.

*2) Your model with Poisson arrival suggests non-linear behavior. You might consider using more sophisticated estimation techniques in future work.*

We have thought of a variety of estimation techniques, but so far have not gained additional insights from either simplification or elaboration. The results in these tables are stable across analytical frameworks. We do have in mind extensions of this research (not for time-wasting, but other play behaviors) that may call for more advanced techniques, so we appreciate your advice.
